# Scenario-based outbreak response training: perspectives from a multidisciplinary trainee team

**DOI:** 10.5365/wpsar.2024.15.5.1116

**Published:** 2024-08-08

**Authors:** Peta Mantel, Shawn Vasoo, Rolando Cruz, Dalva De Assis, Abdurrahman Amin Faisal, Humberto Jaime, Komal Raj Rijal, Sharon Salmon, Jocelyne M Basseal

**Affiliations:** a Indo-Pacific Centre for Health Security, Department of Foreign Affairs and Trade, Canberra, Australia.; b Department of Infectious Diseases, Tan Tock Seng Hospital, Singapore.; c National Centre for Infectious Diseases, Singapore.; d Quezon City Health Department, Manila, Philippines.; e South America Regional Network, Training Programs in Epidemiology and Public Health Interventions Network (TEPHINET), Brasília, Distrito Federal, Brazil.; f Tropical Medicine Research Centre, University of Brasília, Distrito Federal, Brazil.; g Directorate of Surveillance and Health Quarantine, Ministry of Health, Jakarta, Indonesia.; h Regional Office for South Asia, United Nations Children’s Fund (UNICEF), Kathmandu, Nepal.; i Central Department of Microbiology, Tribhuvan University, Kirtipur, Kathmandu, Nepal.; j World Health Organization Regional Office for the Western Pacific, Manila, Philippines.; k UNSW Medicine and Health, School of Population Health, University of New South Wales, Sydney, Australia.; l Sydney Infectious Diseases Institute, Faculty of Medicine and Health, University of Sydney, Sydney, Australia.

The World Health Organization’s (WHO) Global Outbreak Alert and Response Network’s (GOARN) multitiered Capacity-Building and Training Programme aims to strengthen public health capacity to respond to global outbreaks of infectious diseases. Tier 1 introduces the essential knowledge and skills needed before deployment and is delivered via online modules as well as a classroom-based workshop (known as Tier 1.5). Tier 2 is an immersive, residential, scenario-driven simulation exercise, and Tier 3 focuses on specialized leadership training for outbreak responders. Participants in Tiers 2 and 3 must complete an application, and selection is competitive. (*1*) This Perspective has been written by participants and a training faculty member in the Tier 2 outbreak scenario training held in New Delhi, India, from 31 October to 5 November 2022, hosted by WHO’s Regional Offices for South-East Asia and the Western Pacific. It provides a general overview of the GOARN outbreak scenario training programme, participants' perspectives and suggestions for future programmes.

The Tier 2 training is designed for mid-level technical experts from GOARN’s partners who have a minimum of 7 years of professional experience, including in national or international responses to outbreaks, and who are committed to deploying on a GOARN field mission. The selection of training participants is a competitive process requiring submission of a curriculum vitae, a letter of personal motivation and institutional support to attend the training from the GOARN focal point at the partner’s organization. Shortlisted candidates are then assessed using a competency-based interview.

Successful participants complete their initial training via the GOARN online learning management system, which includes modules about GOARN, (*2*) working with GOARN in the field, the landscape of public health emergencies and humanitarian interventions, working as part of an international outbreak response team composed of professionals from different disciplines, personal well-being for deployment and the United Nations’ BSAFE security awareness training. The online training is easy to navigate and provides a useful introduction to the Tier 2 training programme.

Upon arriving for the residential component, participants are divided into three multidisciplinary teams whose members work together throughout the training to determine the agent causing the outbreak in the scenario presented.

During the training, our team (Team Zuum) consisted of eight participants from six countries (Australia, Brazil, Indonesia, Nepal, the Philippines and Singapore) with expertise in epidemiology, clinical management, infection prevention and control, laboratory, and risk communication and community engagement. Three of the eight participants had international deployment experience.

Mirroring the composition of the teams, training faculty are also from different countries and disciplines, anchored by training leads experienced in GOARN deployments and WHO missions. To simulate the work of outbreak response teams, participants are not provided with a training agenda, which contributes to an authentic experience.

Central to the training is the emphasis on developing field skills – that is, the personal qualities and skills necessary to work as part of a team and adapt to various contexts and settings. An evolving scenario incorporating role plays, news reports, field investigations and data analysis forces teams to think creatively, pivot quickly to make decisions and consider the audience for any engagement. Participants in Team Zuum who had deployment experience commented on the realistic nature of the training model, which included the types of intense time and fatigue pressures routinely faced during an outbreak response mission.

Daily mentor-facilitated debriefs with each team provided an opportunity for self-reflection for participants and to improve team dynamics. This helped teams to rapidly move through the stages of team development that would usually occur over a longer period during a deployment.

Despite the diverse and multidisciplinary composition of Team Zuum, no team member had a background in animal health or One Health to complement the expertise in human health. However, as with real-life deployments, team composition is driven by applicants and their availability, so the absence of a One Health expert provided a developmental opportunity for the team to use existing networks to overcome the gap and to understand that deploying with a full complement of technical expertise is not always achievable. The benefit of using a One Health approach has been highlighted in the GOARN Strategy 2022–2026, so participation by One Health experts may increase in the future. (*3*)

## The way forward

While the GOARN Tier 2 training model is highly successful and achieves the desired outcome of preparing technical experts to deploy with GOARN, there is an opportunity to further ongoing coordinated engagement, such as by including alumni among trainees and those being deployed. This engagement may provide support for trainees considering applying for deployment, as alumni can share their lived experiences, connect trainees and those who have been deployed with technical experts, and provide mentoring to less experienced individuals. The strong focus on team-building during Tier 2 training creates enduring links between trainees, which often continue long after the training. Members of Team Zuum have maintained contact through informal channels, building additional relationships within the team and among other teams, faculty members and those who have been previously deployed through formal and informal mechanisms, and these may also prove beneficial in ensuring the longer-term commitment and availability of trainees. A variety of mechanisms could be used to ensure ongoing engagement, such as newsletters and mentoring networks or webinars. Another option is to develop communities of interest to assist in linking technical experts across courses so that networks and relationships can be established before real-life deployments occur. Communities of interest could be based on technical disciplines as defined by GOARN and could also be used to provide a reach-back service for advice during deployments to support technical experts in the field.

Tier 2 training should continue to strengthen and incorporate concepts of adult and experiential learning theory (*4*, *5*) and assess the outcomes of the training with effective evaluation tools that measure how participants’ new skills are applied and their impact. (*6*) GOARN could consider developing further training scenarios that can be delivered in person or virtually to alumni of the Tier 2 trainings to maintain engagement and ensure their skills are current. While GOARN Tier 2 learning is realistic and allows team learning in a simulated environment, it remains a means to an end: actual, successful GOARN deployments.

Intense simulation exercises provide an invaluable training experience for those who will be deployed by GOARN and an opportunity to expand the global network of ready-to-deploy experts. Overall, the Tier 2 training programme is strongly recommended for public health professionals wanting to be better prepared to deploy on an international outbreak response mission with GOARN.
